# A novel human iNeuron tauopathy model identifies splicing and stress granule related RNA binding proteins as regulators of tau aggregation and toxicity

**DOI:** 10.1002/alz70855_099419

**Published:** 2025-12-23

**Authors:** Sarah Kaufman, Jannis Bücking, Rudra Bose, Sarah Hoyle, Abi Wee, Man Wang, Gregory Merz, Daniel Southworth, William W. Seeley, Martin Kampmann

**Affiliations:** ^1^ University of California San Francisco, San Francisco, CA, USA; ^2^ Heidelberg University, Heidelberg, Baden‐Württemberg, Germany

## Abstract

**Background:**

Tauopathies including Alzheimer's disease (AD) and progressive supranuclear palsy (PSP) are characterized by the accumulation of aggregated tau protein in selectively vulnerable cell types that are lost in these diseases. The mechanisms that underlie tau aggregation and its associated toxicity are not known. While several human induced pluripotent stem cell (iPSC)‐derived neuron tauopathy models have been developed, these models do not readily accumulate higher‐order tau aggregates, which has hindered their use when studying mechanisms of tau aggregation and toxicity.

**Methods:**

I created a novel iNeuron tauopathy model system using an established human iPSC line (WTC11) that stably expresses 2N4R(P301S)‐tau fused to mClover3/mApple ("FL‐tau"). At baseline, FL‐tau in control iPSCs and iNeurons remains soluble. However, transduction of tau aggregates originally derived from PSP subject brain lysate induces aggregation of FL‐tau that is stably propagated in iPSCs and maintained through differentiation into iNeurons.

**Results:**

Bulk proteomics of PSP seeded FL‐tau iNeurons versus control FL‐tau iNeurons demonstrated enrichment of several splicing related RNA binding proteins (RBPs), including the nuclear speckle related protein pinin (PNN). While typically localized to the nucleus in non‐dividing iNeurons, nuclear PNN staining was reduced in aggregate‐containing iNeurons and co‐localized with cytoplasmic FL‐tau inclusions. Human neuropathologic analysis further showed that PNN mis‐localizes to neuronal tau aggregates in AD and PSP, and nuclear PNN staining was reduced in these diseases compared to control subjects. CRISPRi mediated knockdown of PNN was toxic in human iPSCs, which further suggests changes in nuclear PNN may contribute to the neuronal loss observed in tauopathies. To identify additional modifiers of tau aggregation, I performed a large‐scale CRISPRi screen of over 5,000 genes in PSP‐seeded iNeurons. This screen identified several novel regulators of tau aggregation, including RBPs related to stress granule formation, RNA maintenance and degradation, and splicing related proteins.

**Conclusions:**

This work identified several novel regulators of tau aggregation and toxicity, including PNN and stress granule related proteins. Future work will examine the mechanism(s) by which these RBPs modulate tau aggregation and toxicity.

